# Functional biomarker signatures of circulating T-cells and its association with distinct clinical status of leprosy patients and their respective household contacts

**DOI:** 10.1186/s40249-020-00763-7

**Published:** 2020-12-20

**Authors:** Pedro Henrique Ferreira Marçal, Rafael Silva Gama, Lorena Bruna Pereira de Oliveira, Olindo Assis Martins-Filho, Roberta Olmo Pinheiro, Euzenir Nunes Sarno, Milton Ozório Moraes, Lucia Alves de Oliveira Fraga

**Affiliations:** 1 Universidade Vale do Rio Doce – Univale, Governador Valadares, MG Brazil; 2 Grupo Integrado de Pesquisas em Biomarcadores, Instituto René Rachou, FIOCRUZ-Minas, Belo Horizonte, MG Brazil; 3 Laboratório de Hanseníase, Instituto Oswaldo Cruz, Fundação Oswaldo Cruz –FIOCRUZ-RJ, Rio de Janeiro, RJ Brazil; 4 Núcleo de Pesquisa em Hansenologia, Universidade Federal de Juiz de Fora, Instituto de Ciências da Vida, Campus Governador Valadares, Rua São Paulo, 582 – Centro, Governador Valadares, MG 30190-002 Brazil

**Keywords:** Leprosy, *Mycobacterium leprae*, Cytokines, Household contacts

## Abstract

**Background:**

Leprosy is a chronic infectious disease classified into two subgroups for therapeutic purposes: paucibacillary (PB) and multibacillary (MB), closely related to the host immune responses. In this context it is noteworthy looking for immunological biomarkers applicable as complementary diagnostic tools as well as a laboratorial strategy to follow-up leprosy household contacts.

**Methods:**

The cross-sectional study enrolled 49 participants, including 19 patients and 30 healthy controls. Peripheral blood mononuclear cells (PBMC) were isolated and incubated in the presence of *Mycobacterium leprae* bacilli. The cells were prepared for surface (CD4^+^ and CD8^+^) and intracytoplasmic cytokine staining (IFN-γ, IL-4 and IL-10). Multiple comparisons amongst groups were carried out by ANOVA, Kruskal–Wallis, Student *T* or Mann–Whitney test. Comparative analysis of categorical variables was performed by Chi-square. Functional biomarker signature analysis was conducted using the global median values for each biomarker index as the cut-off edge to identify the proportion of subjects with high biomarker levels.

**Results:**

The cytokine signature analysis demonstrated that leprosy patients presented a polyfunctional profile of T-cells subsets, with increased frequency of IFN-γ^+^ T-cell subsets along with IL-10^+^ and IL-4^+^ from CD4^+^ T-cells, as compared to health Controls (Venn diagram report). Moreover, statistical analysis was carried out using parametric or non-parametric variance analysis followed by pairwise multiple comparisons, according to the data normality distribution. L(PB) displayed a polyfunctional profile characterized by enhanced percentage of IFN-γ^+^, IL-10^+^ and IL-4^+^ produced by most T-cell subsets, as compared to L(MB) that presented a more restricted cytokine functional profile mediated by IL-10^+^ and IL-4^+^ T-cells with minor contribution of IFN-γ produced by CD4^+^ T-cells. Noteworthy was that HHC(MB) exhibited enhanced frequency of IFN-γ^+^ T-cells, contrasting with HHC(PB) that presented a cytokine profile limited to IL-10 and IL-4.

**Conclusions:**

Our data demonstrated that L(PB) displayed enhanced percentage of IFN-γ^+^, IL-10^+^ and IL-4^+^ as compared to L(MB) that presented functional profile mediated by IL-10^+^ and IL-4^+^ T-cells and HHC(MB) exhibited enhanced frequency of IFN-γ^+^ T-cells, contrasting with HHC(PB). Together, our findings provide additional immunological features associated with leprosy and household contacts. These data provide evidence that biomarkers of immune response can be useful complementary diagnostic/prognostic tools as well as insights that household contacts should be monitored to access putative subclinical infection.

## Background

Leprosy is a chronic infectious disease caused by *Mycobacterium leprae* or *Mycobacterium lepromatosis*, that represents a serious public health problem with more than 200 000 cases reported worldwide [1, 2]. The disease is characterized by skin and peripheral nerves lesions leading to a broad range of clinical manifestations. Leprosy patients can be classified based on the number of skin lesions as paucibacillary (PB) or multibacillary (MB) [3, 4].

The early diagnosis and prompt initiation of treatment are key strategies for leprosy control. Currently the diagnosis of leprosy is achieved essentially by clinical evaluation. Laboratorial methods may also be used to support leprosy diagnosis. However, in general, the laboratorial test displays low sensitivity or low specificity and some methods are difficult to be used outside reference services [5]. To improve leprosy diagnosis performance, it is still necessary to develop and integrate sensitive and specific approaches [6, 7]. In this sense, the search of complementary laboratorial biomarkers is relevant to identify novel targets for leprosy diagnosis. Besides that, the development of diagnostic tools to detect putative subclinical leprosy in household contacts is also crucial to control the transmission of *M. leprae*. It has been proposed that leprosy patients and household contacts share similar profiles of several immunological biomarkers [7] and therefore, the frequency and the size of the daily antigenic exposure may play an important role in the development of leprosy.

The analysis of risk factors have demonstrated that elevated bacillary load in the index case is a relevant variable associated with the development of leprosy amongst the household contacts [8] Besides that, the host immune response may contribute to the clinical outcome of leprosy [4]. It has been demonstrated that *M. leprae* antigens can discriminate contacts and leprosy patients from healthy controls, especially when the controls come from an area non-endemic for leprosy [3, 9]. There are several immunological events that seem to influence the outcome of leprosy, including mechanisms mediated by CD8^+^ and CD4^+^ T-cells [10, 11]. Evaluation of the cell-mediated immune response based on dichotomic cytokine profiles of T-cell subsets has been used to discriminate leprosy clinical presentation [11]. Moreover, it has been proposed that the degree of activation of CD4^+^ and CD8^+^ T-lymphocytes can also allow the discrimination between leprosy patients and their household contacts [7].

Strategies based on biomarkers have been extensively studied in recent years. The main goal of the present study was to characterize the global cytokine signatures of CD4^+^ and CD8^+^ T-cells from leprosy patients with distinct clinical forms and their respective household contacts (HHC) upon in vitro antigen-specific stimuli.

The panoramic cytokine profiles may offer additional insights into the immunological events that are relevant for future clinical studies and patient management.

## Methods

### Subjects, material and methods

#### Ethics statement

This study was approved by the Ethics Committee of the Vale do Rio Doce University (UNIVALE), filed under N˚ PQ 022/09-009. All participants signed a free and informed consent (IC) at the first evaluation. Parents/guardians provided consent on behalf of participants who were minors.

#### Study design and participants

The cross-sectional study was carried out in Governador Valadares, eastern of Minas Gerais State, Brazil, that is considered a hyper endemic area for leprosy with approximately 1.9 cases/10 000 inhabitants compared to Minas Gerais (0.5/10 000) and Brazil (1.2/10 000) [1]. The study enrolled 49 participants of both genders (from 11 to 77 years old), including 19 patients (12 males and 7 females, median age = 37 years old, ranging from 11 to 77 years old) and 30 healthy controls, including nine healthy non-house hold and 21 house-hold contacts (15 males and 15 females, median age = 32 years old, ranging from 13 to 58 years old). According to the Brazilian Guidelines [12], the patients were further categorized into two subgroups referred as: L(PB), (*n* = 9)—patients with tuberculoid-tuberculoid (*n* = 8); borderline-tuberculoid clinical form (*n* = 1), including 5 males and 4 females, median age = 31 years old, ranging from 11 to 64 years old; and L(MB), (*n* = 10)—patients presenting borderline-borderline (*n* = 3); borderline-lepromatous (*n* = 2); lepromatous-lepromatous clinical forms (*n* = 5), including 7 males and 3 females, median age = 42 years old, ranging from 21 to 77 years old. The group of household contacts (HHC) comprised individuals who lives or has lived with a leprosy patient. The HHC group was further categorized into two subgroups as they were exposed to L(PB) or L(MB) patients, and referred as: HHC(PB), (*n* = 11), including 4 males and 7 females, median age = 32 years old, ranging from 13 to 54 years old) and HHC(MB), (*n* = 10), comprising 5 males and 5 females, median age = 30 years old, ranging from 13 to 53 years old). Heparinized whole blood sample was collected from all leprosy patients before chemoterapy treatment and also from healthy controls and used to quantify the intracytoplasmic cytokine profile of lymphocytes upon short-term in vitro culture. *Short-term culture* in vitro *of peripheral blood mononuclear cells.*

Peripheral blood mononuclear cells (PBMC) were isolated from heparinized whole blood samples by Ficoll-Hypaque gradient centrifugation (Pharmacia Fine Chemicals, Piscataway, NJ, USA) and cultured as described previously by Antas [13]. Briefly, three aliquots of 1.0 × 10^6^ PBMC/well were incubated in 24-well flat-bottom plates (Costar Corp., Cambridge, MA, USA) with 1 ml of culture medium (RPMI1640 supplemented) and incubated. In parallel batches, triplicates of 1.0 × 10^6^ PBMC/well were incubated in the presence of sonicated fraction of irradiated *M. leprae* bacilli (10 bacilli/PBMC). *M. leprae*-stimulated and non-stimulated cultures were pre-incubated prior the addition of 3 µg/ml of purified anti-human CD28 antibody (Pharmingen Inc., San Diego, CA, USA) for 1 h and the addition of 10 µg/ml of Brefeldin A (Sigma Immunochemical, St. Louis, MO, USA) 4 h before the end of incubation achieved at 16, 20 and 24 h. Positive control cultures were carried out using 50 ng/ml PMA plus 1 µg/ml ionomycin (Sigma Immunochemical, St. Louis, MO, USA) to confirm cell viability. After incubation, cells were harvested and prepared for surface and intracytoplasmic cytokine staining according to Teixeira-Carvalho [14], modified as follows: cultured PBMC suspensions were adjusted to 5.0 × 10^5^ cells/ml and incubated with anti-CD4/PercP and anti-CD8/PercP-Cy5.5 (BD Bioscience, San Diego, CA, USA). Following, cells were washed fixed in 4% paraformaldehyde (PFA) and permeabilized with 0.3% saponin. After fix/perm procedures the cells were stained with anti-IFN-γ, anti-IL-4 or anti-IL-10, labeled with PE (Pharmingen Inc., San Diego, CA, USA). After two wash steps, stained cells were suspended with 1% PFA and immediately analyzed in a EPICS MCL® flow cytometer (BD Bioscience, San Jose, CA, USA) equipped with the CellQuest® software. A total of 50 000 events gated on lymphocyte regions were collected per sample. Distinct gating strategies were employed to quantify the percentages of cytokine^+^ cells within CD4^+^ and CD8^+^ T-cells. The percentages of cytokine^+^ T-cells were estimated as the sum of cytokine-producing CD4^+^ and CD8^+^ cells. The results were expressed as mean frequency (‰) ± standard error of cytokine^+^ cells amongst gated lymphocyte subsets upon in vitro culture in the absence/presence of *M. leprae* antigen.

### Data analysis

Multiple comparisons amongst groups were carried out by ANOVA and Kruskal–Wallis test followed by Tuckey or Dunn’s post-test for sequential pairwise comparisons. Additionally, Student *t* or Mann–Whitney test were also employed for pairwise comparative analysis. Comparative analysis of categorical variables was carried out by Chi-square. In all cases, significant differences were considered at *P* < 0.05. The Graph Pad Prism software (Version 5.0, Graph Pad Software, San Diego, CA, USA) was employed to perform all statistical analyses and graphical arts.

Functional biomarker signature analysis was carried out as previously reported by Vitelli-Avelar et al. [15]; Silva et al. [16] and Mota et al. [17], modified as follow. The global median values were used for each biomarker index (*M. leprae*-stimulated/non-stimulated culture) as the cut-off edge to identify the proportion of subjects with high biomarker levels, i.e. above the global median cut-off. The descriptive establishment of cut-offs are provided in the Additional file 1: Fig. S1. The cut-off values employed comprised: (IFN-γ^+^ T-cells = 1.0, IL-4^+^ T-cells = 0.9 and IL10^+^ T-cells = 1.1; IFN-γ^+^ CD4^+^ = 1.0, IL-4^+^ CD8^+^ -4 = 0.8 and IL-10^+^ CD4^+^ = 0.9; IFN-γ^+^ CD8^+^ = 1.0, IL-4^+^ CD8^+^ = 0.8 and IL-10^+^ CD8^+^ = 1.0). Those biomarkers with more than 50% of subjects above the cut-off were underscored and considered for comparative analysis amongst groups. Overlaid ascendant biomarker signatures were employed for comparative analysis amongst groups. The major advantages of using this approach to describe the cytokine signature is its ability to detect, with higher sensibility, putative minor changes not detectable by conventional statistical methods. Venn diagram analysis (https://bioinformatics.psb.ugent.be/webtools/Venn/) was employed to identify biomarkers selectively observed in Leprosy patients as compared to Healthy Controls as well as in Household Contacts and Leprosy subgroups.

## Results

### Analysis of cytokine^+^ cells amongst peripheral blood mononuclear cells upon in vitro culture in the presence/absence of ***M. leprae*** antigen

**Fig. 1:**
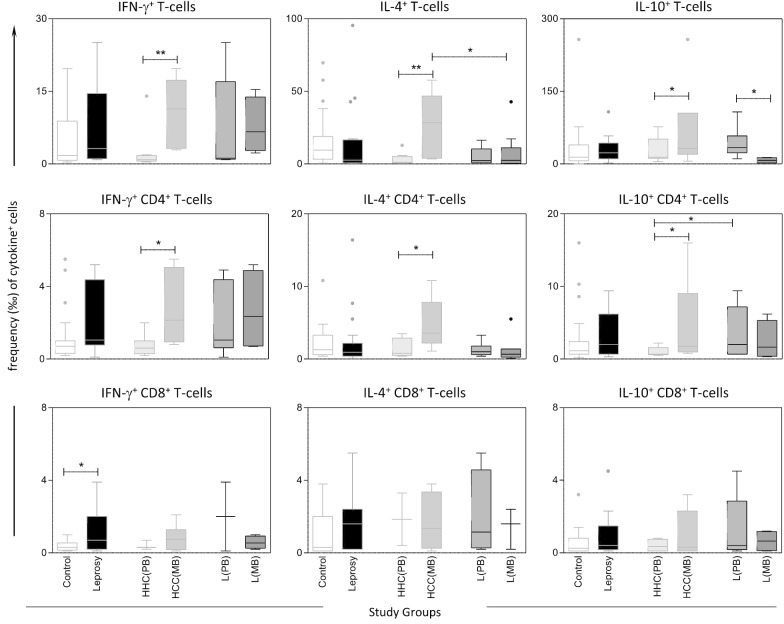
Frequency of cytokine^+^ cells in Leprosy patients, Household Contacts and Healthy Controls upon in vitro non-stimulated culture. The frequency (‰) of cytokine^+^ cells (IFN-γ, IL-4 and IL-10) amongst gated lymphocyte subsets including: T-cells, CD4^+^ T-cells and CD8^+^ T-cells were calculated upon in vitro culture in the absence of exogenous stimuli (non-stimulated culture). The results are presented in boxplot format, indicating the median values (min–max) and the outliers underscored by dots, based on Tuckey analysis for Healthy Controls =  ; Leprosy patients =  ; HHC(PB) =  —Household Contacts of Paucibacillary Leprosy patients; HHC(MB) =  —Household Contacts of Multibacillary Leprosy patients; L(PB) =  —Paucibacillary Leprosy patients; L(MB) =  —Multibacillary Leprosy patients. Multiple comparisons amongst groups were carried out by Kruskal–Wallis test followed by Dunn’s post-test for sequential pairwise comparisons. Additionally, Man-Whitney Test were also employed for pairwise comparative analysis. Significant differences, using the above-mentioned statistical methods are underscored by * for differences at *P* < 0.05 and ** for differences at *P* < 0.01

**Fig. 2:**
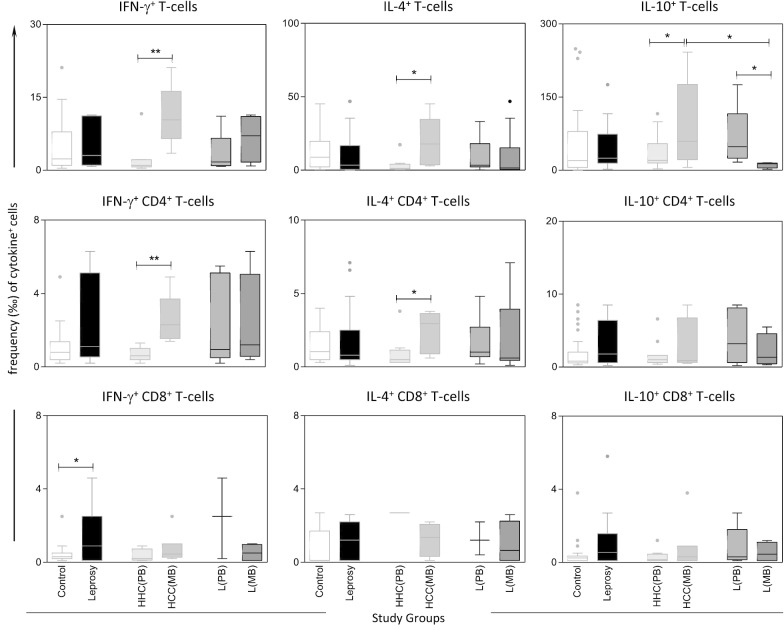
Frequency of cytokine^+^ cells in Leprosy patients, Household Contacts and Healthy Controls upon in vitro* M. leprae*-stimulated culture. The frequency (‰) of cytokine^+^ cells (IFN-γ, IL-4 and IL-10) amongst gated lymphocyte subsets including: T-cells, CD4^+^ T-cells and CD8^+^ T-cells were calculated upon in vitro culture in the absence of exogenous stimuli (non-stimulated culture). The results are presented in boxplot format, indicating the median values (min–max) and the outliers underscored by dots, based on Tuckey analysis for Healthy Controls =  ; Leprosy patients =  ; HHC(PB) =  —Household Contacts of Paucibacillary Leprosy patients; HHC(MB) =  —Household Contacts of Multibacillary Leprosy patients; L(PB) =  —Paucibacillary Leprosy patients; L(MB) =  —Multibacillary Leprosy patients. Multiple comparisons amongst groups were carried out by Kruskal–Wallis test followed by Dunn’s post-test for sequential pairwise comparisons. Additionally, Man-Whitney Test were also employed for pairwise comparative analysis. Significant differences, using the above-mentioned methods, are underscored by * for differences at *P* < 0.05 and ** for differences at *P* < 0.01

Short-term cultures in vitro of peripheral blood mononuclear cells from leprosy patients and healthy Controls were carried out to quantify the percentages of cytokine^+^ cells (IFN-γ, IL-4 and IL-10) amongst T-cells, CD4^+^ T-cells and CD8^+^ T-cells. The results presented in Fig. 1 and Fig. 2. The Additional file 2: Table S1 presented detailed analysis of these findings.

Data analysis was carried out by variance analysis followed by post-test for sequential pairwise comparisons and the results demonstrated that Leprosy patients presented higher frequency of IFN-γ^+^ CD8^+^ T-cells as compared to healthy controls (*P* = 0.02; *P* = 0.04, respectively), for non-stimulated (Fig. 1) and *M. leprae*-stimulated culture (Fig. 2). Moreover, HHC (MB) exhibited higher frequency of IFN-γ^+^, IL-4^+^ and IL-10^+^ events amongst T-cells in both non-stimulated (*P* = 0.008; *P* = 0.009; *P* = 0.05, respectively) or *M. leprae*-stimulated culture (*P* = 0.003; *P* = 0.02; *P* = 0.05, respectively) as compared to HHC(PB). The frequencies of IFN-γ^+^, IL-4^+^ and IL-10^+^ CD4^+^ T-cells in non-stimulated culture were higher in HHC (MB) (*P* = 0.01; *P* = 0.02; *P* = 0.05, respectively) as compared to HHC (PB) (Fig. 1, Additional file 2: Table S1). Furthermore, in *M. leprae*-stimulated culture, the HHC (MB) showed high frequency of IFN-γ^+^ and IL-4^+^ amongst CD4^+^ T-cells (*P* = 0.007; *P* = 0.03, respectively) as compared to HHC (PB) (Fig. 2, Additional file 2: Table S1). Additionally, L (MB) displayed a decreased frequency of IL-10^+^ T-cells (*P* = 0.05; *P* = 0.01), in the non-stimulated (Fig. 1, Additional file 2: Table S1) and *M. leprae*-stimulated culture (Fig. 2, Additional file 2: Table S1), respectively, as compared to L(PB).

Complementary analysis of biomarker indexes (*M. leprae*-stimulated/non-stimulated culture) further demonstrated that L(MB) showed lower levels of IL-10^+^ CD8^+^ T-cells as compared to L(PB) (*P* = 0.05) (Table 1).

**Table 1:** Cytokine profile amongst peripheral blood mononuclear cells from leprosy patients, household contacts and healthy controls upon in vitro culture

Parameters	Groups		Subgroups			
	Healthy Controls	Leprosy	HHC		Leprosy	
			HHC(PB)	HHC(MB)	L(PB)	L(MB)
*Mycobacterium leprae*-stimulated /Non-stimulated Culture INDEX*						
T-cells						
IFN-γ^+^	1.4 ± 0.2	1.1 ± 0.2	1.3 ± 0.2	1.3 ± 0.4	1.3 ± 0.2	0.8 ± 0.2
IL-4^+^	1.3 ± 0.2	1.0 ± 0.2	1.6 ± 0.6	0.8 ± 0.1	1.0 ± 0.2	1.0 ± 0.2
IL-10^+^	2.3 ± 0.7	2.3 ± 0.8	2.1 ± 0.7	3.6 ± 2.0	2.2 ± 0.9	2.5 ± 1.6
CD4^+^T-cells						
IFN-γ^+^	1.3 ± 0.2	1.0 ± 0.2	1.3 ± 0.3	1.5 ± 0.3	1.1 ± 0.2	0.9 ± 0.3
IL-4^+^	0.8 ± 0.1	1.2 ± 0.3	0.7 ± 0.1	0.7 ± 0.1	0.9 ± 0.2	1.4 ± 0.5
IL-10^+^	1.1 ± 0.2	1.5 ± 0.5	1.1 ± 0.2	1.1 ± 0.3	1.2 ± 0.2	1.9 ± 1.4
CD8^+^T-cells						
IFN-γ^+^	1.2 ± 0.2	0.9 ± 0.2	1.0 ± 0.2	1.2 ± 0.4	1.3 ± 0.1	0.8 ± 0.2
IL-4^+^	0.8 ± 0.1	0.7 ± 0.1	0.8 ± 0.0	1.0 ± 0.2	0.6 ± 0.1	0.8 ± 0.2
IL-10^+^	1.1 ± 0.2	1.1 ± 0.1	1.1 ± 0.1	0.9 ± 0.2	1.2 ± 0.1	0.8 ± 0.1^e^

*HHC(PB)* household contacts of paucibacillary leprosy patients, *HHC(MB)* household contacts of multibacillary leprosy patients, *L(PB)* paucibacillary leprosy patients, *L(MB)* multibacillary leprosy patients

*Data are expressed as mean INDEX (‰) ± standard error of cytokine^+^ cells (IFN-γ, IL-4 and IL-10) amongst gated lymphocyte subsets upon in vitro culture in the presence/absence of *M. leprae* antigen, including: T-cells, CD4^+^ T-cells and CD8^+^ T-cells. Multiple comparisons amongst groups were carried out by ANOVA test followed by Tuckey post-test for sequential pairwise comparisons. Additionally, Student *t* test were also employed for pairwise comparative analysis. Significant differences at *P* < 0.05, using the above-mentioned statistical methods is underscored by the letter “e” for comparison with L(PB)

### Antigen-specific cytokine signature of peripheral blood mononuclear cells upon in vitro culture in the presence/absence of *M. leprae* antigen

The antigen-specific cytokine signature was assembled for each parameter, including IFN-γ, IL-4 and IL-10-producing T-cells, CD4^+^ T-cells and CD8^+^ T-cells. Categorical transformation was carried out using the global median values for each biomarker as the cut-off to identify subjects with high biomarker indexes. The results presented in Table 2.

**Table 2:** Antigen-specific cytokine profile of peripheral blood mononuclear cells from leprosy patients, household contacts and healthy controls

Parameters	Groups		Subgroups			
	Healthy controls	Leprosy	HHC		Leprosy	
			HHC(PB)	HHC(MB)	L(PB)	L(MB)
*Proportion of subjects with biomarker INDEX above the global median cut-off*^#^						
T-cells						
IFN-γ^+^	46 (11/24)	50 (05/10)	44 (04/09)	50 (03/06)	67 (04/06)	25 (01/04)
IL-4^+^	50 (12/24)	47 (08/17)	44 (04/09)	33 (02/06)	57 (04/07)	40 (04/10)
IL-10^+^	47 (14/30)	55 (06/11)	64 (07/11)	50 (05/10)	57 (04/07)	50 (02/04)
CD4^+^T-cells						
IFN-γ^+^	42 (10/24)	50 (05/10)	44 (04/09)	67 (04/06)	50 (03/06)	50 (02/04)
IL-4^+^	39 (09/23)	69 (11/16)^a^	33 (03/09)	20 (01/05)	57 (04/07)	78 (07/09)^e^
IL-10^+^	47 (14/30)	73 (08/11)	55 (06/11)	40 (04/10)	86 (06/07)	50 (02/04)
CD8^+^T-cells						
IFN-γ^+^	39 (07/18)	50 (03/06)	33 (01/03)	33 (02/06)	100 (02/02)^f^	25 (01/04)
IL-4^+^	78 (07/09)	17 (01/06)^a^	100 (01/01)	75 (03/04)	0 (00/03)^d^	33 (01/03)
IL-10^+^	31 (05/16)	30 (03/10)	25 (01/04)	29 (02/07)	50 (03/06)	0 (00/04)^e^

^#^Data are expressed as proportion (%) of subjects with biomarker INDEX (*M. leprae*-stimulated/non-stimulated culture) above the Global Median cut-off, established for each cytokine^+^ cells (IFN-γ, IL-4 and IL-10) amongst gated lymphocyte subsets upon in vitro culture in the presence/absence of *M. leprae* antigen, including: T-cells, CD4^+^ T-cells and CD8^+^ T-cells. Comparative analysis of was carried out by Chi-square. Significant differences at *P* < 0.05, using the above-mentioned statistical methods is underscored by letters “a”, “d”, “e” and “f” in comparisons to Controls, HHC(MB), L(PB) and L(MB), respectively

*HHC(PB)* household contacts of paucibacillary leprosy patients, *HHC(MB)* household contacts of multibacillary leprosy patients, *L(PB)* paucibacillary leprosy patients, *L(MB)* multibacillary leprosy patients

Statistical analysis was carried out using parametric or non-parametric variance analysis followed by pairwise multiple comparisons, according to the data normality distribution. It was demonstrated that the Leprosy group comprises a higher proportion of subjects with enhanced levels of IL-4^+^ CD4^+^ T-cells (*P* = 0.05) as compared to health Controls. Conversely, the Leprosy group exhibited a decrease proportion of subjects with IL-4^+^ CD8^+^ T-cells (*P* = 0.02) above the global median cut-off as compared to health Controls. No significant differences were observed between subgroups of Household contacts. Comparative analysis between subgroups of Leprosy patients showed that L(PB) presented a higher proportion of IFN-γ^+^ CD8^+^ T-cells (*P* = 0.046) and lower proportion of IL-4^+^ CD8^+^ T-cells (*P* = 0.02) as compared to L(MB). On the other hand, L(MB) group presented higher proportion of IL-4^+^ CD4^+^ T-cells (*P* = 0.043) and lower frequency of IL-10^+^ CD8^+^ T-cells (*P* = 0.05) as compared to L(PB) (Table 2).

### Functional biomarker signatures in leprosy patients and healthy controls

**Fig. 3:**
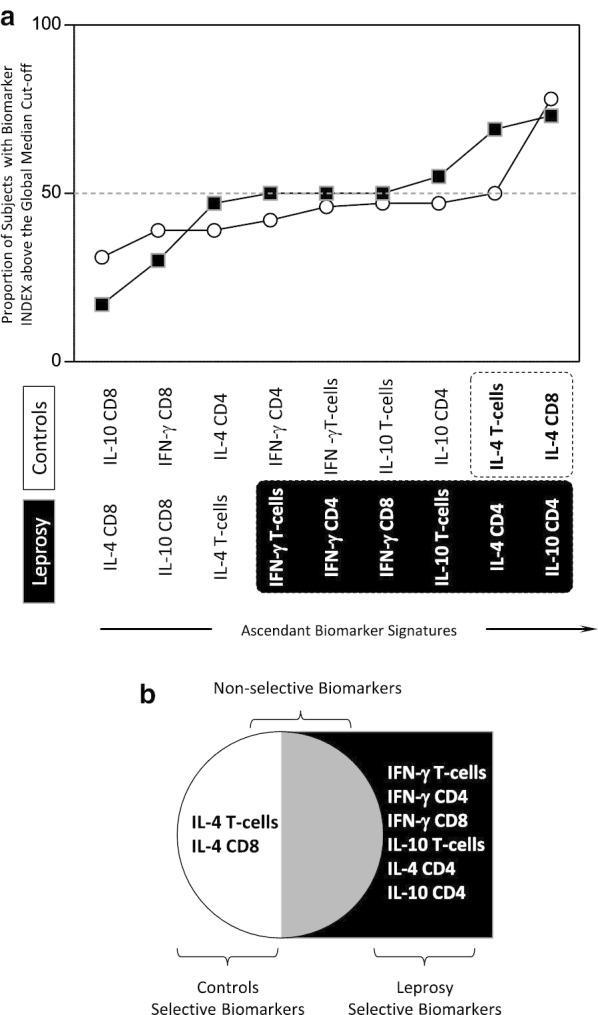
Functional biomarker signatures in Leprosy patients and Healthy Controls. **a** Overlaid biomarker signatures of Leprosy patients (  ) and Controls (  ) were assembled to select biomarkers with proportion of subjects above the global median cut-off higher than the 50% in each group (black/white background rectangles). **b** Venn diagram report was employed to identify the set of biomarkers selectively increased in Leprosy patients as compared to Healthy Controls. These attributes were tagged in bold format

The comparative analysis of ascendant biomarker signatures between leprosy patients and healthy controls is presented in Fig. 3. Overlaid biomarker signatures were assembled to select biomarkers with proportion of subjects above the global median cut-off higher than the 50% in each group. These attributes were tagged for further analysis (Fig. 3a).

Venn diagram report was employed to identify the set of biomarkers selectively increased in leprosy patients as compared to healthy controls. The analysis of Venn diagram report allowed the selection of IFN-γ^+^ T-cells, IFN-γ^+^ CD4^+^ T-cells, IFN-γ^+^ CD8^+^ T-cells, IL-10^+^ T-cells, IL-4^+^ CD4^+^ T-cells and IL-10^+^ CD4^+^ T-cells as Leprosy-selective biomarkers. Moreover, the attributes IL-4^+^ T-cells and IL-4^+^ CD8^+^ T-cells were identified as healthy Controls-selective biomarkers (Fig. 3b).

### Functional biomarker signatures in subgroups of household contacts and leprosy patients with distinct clinical forms

**Fig. 4:**
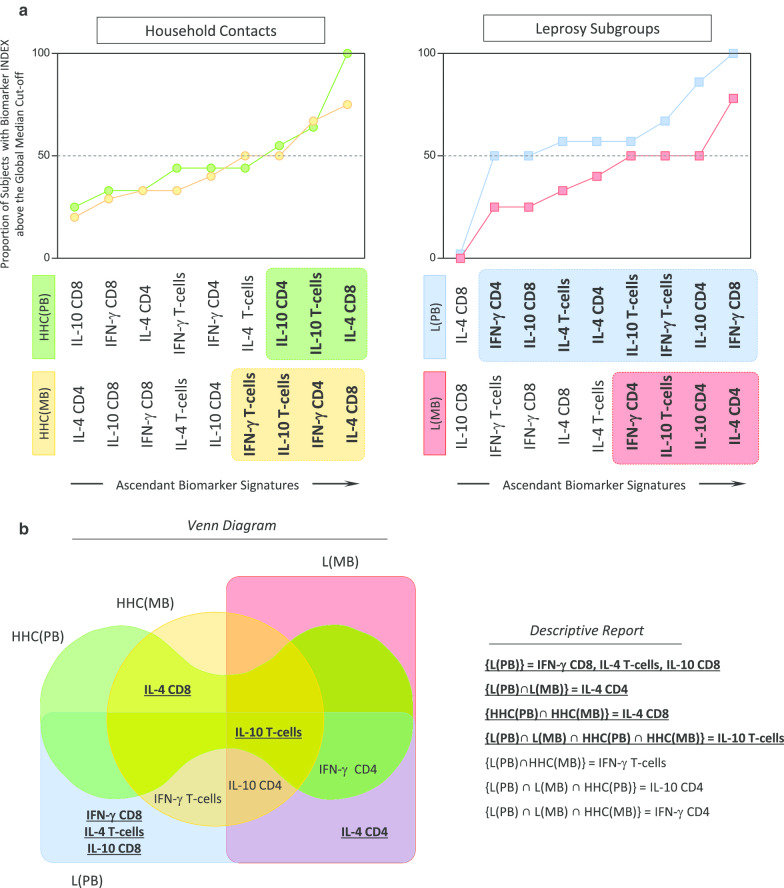
Functional biomarker signatures in subgroups of Leprosy patients and Household contacts. **a** Overlaid biomarker signatures of subgroups of Household contacts (HHC(PB) =  and HHC(MB) =  ) and subgroups of Leprosy patients (L(PB) =  and L(MB) =  ) were assembled to select biomarkers above the global median cut-off with proportion higher than the 50% in each group (black/white background rectangles). These attributes were tagged in bold format. **b** Venn diagram report was employed to identify the set of biomarkers selectively increased in each subgroup of Household contacts and subgroups of Leprosy patients. These attributes were highlighted in bold underline format

Comparative analyses of ascendant biomarker signatures between subgroups of Household contacts and subgroups of Leprosy patients presented in Fig. 4. Overlaid biomarker signatures were assembled to select biomarkers with proportion of subjects above the global median cut-off higher than the 50% in each group. Data analysis demonstrate that the attributes IL-10^+^ CD4^+^ T-cells, IL-10^+^ T-cells and IL-4^+^ CD8^+^ T-cells were increased in HHC (PB), whereas IFN-γ^+^ T-cells, IL-10^+^ T-cells, IFN-γ^+^ CD4^+^ T-cells and IL-4^+^ CD8^+^ T-cells were highlighted in HHC (MB). Furthermore, the attributes IFN-γ^+^ CD4^+^ T-cells, IL-10^+^ CD8^+^ T-cells, IL-4^+^ T-cells, IL-4^+^ CD4^+^ T-cells, IL-10^+^ T-cells, IFN-γ^+^ T-cells, IL-10^+^ CD4^+^ T-cells and IFN-γ^+^ CD8^+^ T-cells were tagged in L (PB), while IFN-γ^+^ CD4^+^ T-cells, IL-10^+^ T-cells, IL-10^+^ CD4^+^ T-cells and IL-4^+^ CD4^+^ T-cells were underscored in L (MB) (Fig. 4a).

Venn diagram report was employed to identify the set of biomarkers selectively increased in each subgroup of Household contacts and subgroups of Leprosy patients. Using this approach, the attribute IL-10^+^ T-cells was identified as a universal biomarker observed in all subgroups. The attribute IL-4^+^ CD8^+^ T-cells was a general biomarker to identify both Household contacts, whereas IL-4^+^ CD4^+^ T-cells was a general biomarker for both subgroups of Leprosy patients. L(PB) selective biomarkers comprise IFN-γ^+^ CD8^+^ T-cells, IL-4^+^ T-cells and IL-10^+^ CD8^+^ T-cells (Fig. 4b).

## Discussion

Leprosy still persists as a relevant public health problem in several countries in Asia, Africa, and Latin America [1]. The host immune response plays a critical role in the pathophysiology of Leprosy, that together with genetic factors influence the clinical course of disease [18].

The use of immunological biomarkers as diagnostic/prognostic tools is a relevant complementary strategy to classify leprosy patients into distinct clinical forms and support early clinical interventions, prompt initiation of treatment and effective leprosy control. It has been proposed that immunological biomarkers can also be a useful laboratorial strategy to monitor household contacts [7]. Queiroz et al. have compared several immunological features of leprosy index cases and their household contacts aiming at identifying biomarkers to monitor household contacts. Although, several immunological parameters displayed similar profile index cases and household contacts, suggesting the lack of immunological distance between them, some biomarkers could be used as laboratorial tools to monitor household contacts. Specifically, increased antibody response, higher levels of lymphocyte activation (expression of CD25 by CD4^+^ and CD8^+^ T-cells) along with enhanced frequency of monocytes circulating in the peripheral blood were observed in leprosy patients as compared to contacts [7]. On the other hand, it has been proposed by Bernard Naafs [19] that there is no specific test to predict who will develop leprosy after exposure. Indeed, the observation that super exposure to *M. leprae* can lead to a decrease in host resistance was first described in 1973 [20]. These authors showed that contacts of lepromatous patients with active disease, displayed lower in vitro responses to *M. leprae* when compared with contacts of lepromatous patients treated for more than six months. In this sense, it has been stated that regardless of the improvements in understanding of the immune responses in leprosy, to date, most studies failed to provide effective and robust methods with predictive applicability. Currently, attempts are still necessary to increase the limited clusters of immune parameters already available for predictive diagnosis and laboratorial monitoring.

In the present study, we have characterized the functional cytokine signatures of CD4^+^ and CD8^+^ T-cells from leprosy patients with distinct clinical forms and their respective household contacts (HHC), using a model of in vitro antigen-specific stimuli for peripheral blood mononuclear cells. Our data demonstrated that leprosy patients presented an overall polyfunctional profile of T-cells subsets, with increased frequency of IFN-γ^+^ T-cell subsets along with IL-10^+^ and IL-4^+^ from CD4^+^ T-cells. According to Queiroz [7] the highest degree of activation of T CD4^+^ and CD8^+^ T-lymphocytes in the index cases, could be, a response to antigenic stimulation by the bacillus, with consequent increased CD86 expression by the monocytes that activate these lymphocytes, generating effector and/or memory T-cells. Differential costimulatory molecule expression by T-cells was also verified by Shibuya et al. [21], among lepromatous and tuberculoid patients and contacts. Molecules that either signal for (CD86 and CD28) or reduce (CD152 and PD-1) T-cell activation were evaluated. Probably as a consequence of defective APC function, T-cells from lepromatous patients were driven to an anergic state and exhibited reduced expression of both CD86 and CD28, especially when compared with tuberculoid patients. Additionally, our results demonstrated that L(PB) displayed a polyfunctional profile characterized by enhanced percentage of IFN-γ^+^, IL-10^+^ and IL-4^+^ produced by most T-cell subsets, as compared to L(MB) that presented a more restricted cytokine functional profile mediated by IL-10^+^ and IL-4^+^ T-cells with minor contribution of IFN-γ produced by CD4^+^ T-cells. Since the multibacillary household contacts are exposed to a larger antigenic stimulus, it may determine together with their adaptive immunity, this overall cytokine profile observed. There is a predominance of a type-2 lymphocytes response in the lepromatous form, which induces the production of cytokines such as IL-4, IL-10, and TGF-β that inactivate the microbicidal response of macrophages, thereby facilitating the survival of the bacillus [22]. Considering the minor contribution of IFN-γ among L(MB), our results corroborate previous studies [23, 24]. Noteworthy was that HHC(MB) exhibited enhanced frequency of IFN-γ^+^ T-cells, contrasting with HHC(PB) that presented a cytokine profile limited to IL-10 and IL-4. The precise relationship between IFN-γ production and *M. leprae* exposure is still controversial. Martins et al. [23] have proposed a direct correlation between the levels of IFN-γ and the degree of exposure to *M. leprae*. According to Sampaio et al., [24] the *M. leprae*-induced IFN-γ production was higher in multibacillary contacts as compared to those paucibacillary contacts. Moreover, the percentage of individuals showing IFN-γ production in response to *M. leprae* antigen stimuli in vitro was higher amongst household and occupational contacts suggesting a high frequency of sensitization. On the other hand, Martins et al. [23], showed that the levels of IFN-γ^+^ production in response to *M. leprae* antigen or *M. leprae*-specific peptides is progressively reduced with increasing exposure to *M. leprae* and further diminished in leprosy patients. It is possible that, the extent of response to *M. leprae* antigen among the contacts primarily depended on the length of contact and degree of infectiousness of the index cases rather than the degree of consanguinity with the index patients [24]. Moreover, it is possible that a balance between IFN-γ and IL-10 may play a role in this scenario. In this sense, Geluk et al. [25], showed that ratio IFN-γ/IL-10 was higher for asymptomatic individuals compared to either leprosy patient. Together, our findings supply additional immunological features associated with leprosy and household contacts. These data provide evidence that biomarkers of immune response can be useful complementary diagnostic/prognostic tools. According to Geluk et al. [25], IFN-γ production induced by *M. leprae*-unique proteins can identify individuals highly exposed to *M. leprae* and therefore more at risk for developing disease and/or transmitting the bacterium.

Previous reports from our group have also demonstrated the presence of *M. leprae* DNA in scrapes and blood samples as well as high levels of antibodies to *M. leprae* recombinant proteins in household contacts [26, 27]. These findings suggest that household contact should be monitored for putative subclinical infection. This hypothesis was further confirmed in a 6 years’ follow-up study demonstrating that a long time some household contacts developed leprosy disease [28]. Therefore, the significant level of antigen-specific cytokine secretion by peripheral blood cells of household contacts, especially those HHC(MB), described in the present investigation, provides reliable evidence of their higher susceptibility to leprosy disease. However, the present study may have some limitations regarding the number of samples evaluated. Further studies are still required to validate these findings in a larger number of samples. Overall, our data support the relevance of investigating elements of the cell-mediated immune response as potential biomarker to monitor household contacts.

## Conclusion

Our data demonstrated that L(PB) displayed enhanced percentage of IFN-γ^+^, IL-10^+^ and IL-4^+^ as compared to L(MB) that presented functional profile mediated by IL-10^+^ and IL-4^+^ T-cells and HHC(MB) exhibited enhanced frequency of IFN-γ^+^ T-cells, contrasting with HHC(PB). Our findings provide evidence that biomarkers of the immune response can be useful complementary diagnostic/prognostic tools as well as insights that household contacts should be closely monitored, especially those HHC (MB), that showed a significant level of antigen-specific cytokine secretion by peripheral blood cells.

Early detection of leprosy cases and effective chemotherapy are the best strategies to reduce the incidence of new cases of leprosy and prevent transmission. Considering that HHCs comprise a group of subjects with a high risk of disease, we suggest that, as a prevention strategy, detection of antigen-specific cytokine secretion should be used to follow-up with leprosy HHCs to confirm or rule out subclinical infection.

## Supplementary information

**Additional file 1: Supplementary figure 1.** Establishment of Cut-offs to segregate subjects with low or high Biomarker INDEX.

**Additional file 2: Supplementary table 1.** Frequency of cytokine^+^ cells amongst peripheral blood mononuclear cells from leprosy patients, household contacts and healthy controls upon in vitro culture.

## Data Availability

All data generated or analysed during this study are included in this published article and its supplementary information files (Additional files 1, 2).

## Abbreviations

BI: Bacilloscopy index
L(PB): Leprosy (paucibacillary)
L(MB): Leprosy multibacillary
HHC(PB): Household contacts of paucibacillary
HHC(MB): Household contacts of multibacillary
CREDENPES: Center for endemic diseases and special programs
IC: Informed consent
*M. leprae*: *Mycobacterium leprae*
N: Number of patients
