# Supplementary material for: Functional biomarker signatures of circulating T-cells and its association with distinct clinical status of leprosy patients and their respective household contacts
Source: Infect Dis Poverty. 2020 Dec 20;9:167. doi: 10.1186/s40249-020-00763-7 (PMC7749990; doi:10.1186/s40249-020-00763-7)
Supplement: Supplementary file 1 — Additional file 1: Supplementary figure 1. Establishment of Cut-offs to segregate subjects with low or high Biomarker INDEX. [file 40249_2020_763_MOESM1_ESM.pptx]

## Slide 1
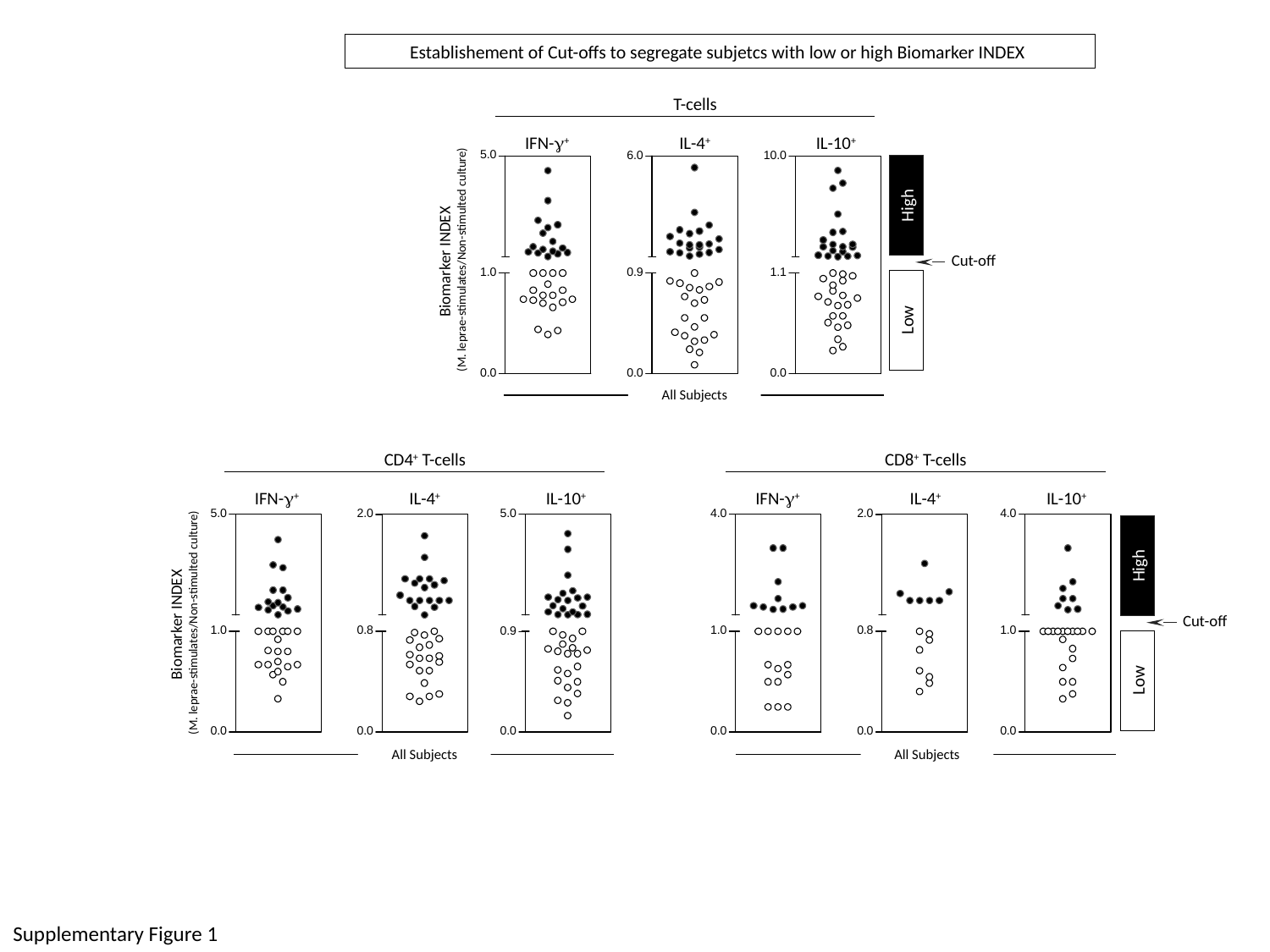

Establishement of Cut-offs to segregate subjetcs with low or high Biomarker INDEX
T-cells
IFN-g+
IL-4+
IL-10+
High
Biomarker INDEX
(M. leprae-stimulates/Non-stimulted culture)
Cut-off
Low
All Subjects
CD4+ T-cells
CD8+ T-cells
IFN-g+
IL-4+
IL-10+
IFN-g+
IL-4+
IL-10+
High
Biomarker INDEX
(M. leprae-stimulates/Non-stimulted culture)
Cut-off
Low
All Subjects
All Subjects
Supplementary Figure 1
