# Supplementary material for: Functional biomarker signatures of circulating T-cells and its association with distinct clinical status of leprosy patients and their respective household contacts
Source: Infect Dis Poverty. 2020 Dec 20;9:167. doi: 10.1186/s40249-020-00763-7 (PMC7749990; doi:10.1186/s40249-020-00763-7)
Supplement: Supplementary file 2 — Additional file 2: Supplementary table 1. Frequency of cytokine+ cells amongst peripheral blood mononuclear cells from leprosy patients, household contacts and healthy controls upon in vitro culture. [file 40249_2020_763_MOESM2_ESM.docx]

Supplementary Table 1. Frequency of cytokine+ cells amongst peripheral blood mononuclear cells from leprosy patients, household contacts and healthy controls upon *in vitro* culture

| **Parameters*** | | |  | **Groups** | | |  | **Subgroups** | | | | | | |
| --- | --- | --- | --- | --- | --- | --- | --- | --- | --- | --- | --- | --- | --- | --- |
|  |  |  |  | **Healthy Controls** |  | **Leprosy** |  | **HHC** | | |  | **Leprosy** | | |
|  |  |  |  |  |  |  |  | **HHC(PB)** |  | **HHC(MB)** |  | **L(PB)** |  | **L(MB)** |
|  |  |  |  |  |  |  |  |  |  |  |  |  |  |  |
| **Non-stimulated Culture** |  | **T-cells** |  |  |  |  |  |  |  |  |  |  |  |  |
|  |  | **IFN-γ^+^** |  | 4.6±1.2 |  | 7.5±2.6 |  | 2.4±1.4 |  | **10.9±2.8^c^** |  | 7.3±4.2 |  | 7.8±2.9 |
|  |  | **IL-4^+^** |  | 15.7±3.8 |  | 13.9±5.8 |  | 3.2±1.5 |  | **27.6±9.0^c,f^** |  | 5.1±2.2 |  | 8.0±4.7 |
|  |  | **IL-10^+^** |  | 43.1±14.8 |  | 30.6±9.3 |  | 29.5±7.4 |  | **88.4±40.8^c^** |  | 43.8±12.0 |  | **7.6±3.0^e^** |
|  |  |  |  |  |  |  |  |  |  |  |  |  |  |  |
|  |  | **CD4^+^T-cells** |  |  |  |  |  |  |  |  |  |  |  |  |
|  |  | **IFN-γ^+^** |  | 1.2±0.3 |  | 2.3±0.6 |  | 0.7±0.2 |  | **2.8±0.8^c^** |  | 2.0±0.8 |  | 2.7±1.1 |
|  |  | **IL-4^+^** |  | 2.2±0.5 |  | 2.6±0.9 |  | 1.5±0.4 |  | **4.7±1.6^c,f^** |  | 3.2±1.9 |  | 1.2±0.6 |
|  |  | **IL-10^+^** |  | 2.5±0.6 |  | 3.2±1.0 |  | 1.0±0.2 |  | **4.7±1.7^c^** |  | **3.6±1.3^d^** |  | 2.5±1.4 |
|  |  |  |  |  |  |  |  |  |  |  |  |  |  |  |
|  |  | **CD8^+^T-cells** |  |  |  |  |  |  |  |  |  |  |  |  |
|  |  | **IFN-γ^+^** |  | 0.4±0.1 |  | **1.2±0.5^a^** |  | 0.4±0.2 |  | 0.8±0.3 |  | 2.0±1.1 |  | 0.6±0.2 |
|  |  | **IL-4^+^** |  | 1.0±0.4 |  | 1.7±0.7 |  | 1.9±1.5 |  | 1.7±0.8 |  | 2.0±1.2 |  | 1.4±0.6 |
|  |  | **IL-10^+^** |  | 1.1±0.6 |  | 1.1±0.4 |  | 0.4±0.2 |  | 1.9±1.1 |  | 1.3±0.7 |  | 0.7±0.3 |
|  |  |  |  |  |  |  |  |  |  |  |  |  |  |  |
|  |  |  |  |  |  |  |  |  |  |  |  |  |  |  |
| ***M. leprae*-stimulated Culture** |  | **T-cells** |  |  |  |  |  |  |  |  |  |  |  |  |
|  |  | **IFN-γ^+^** |  | 4.6±1.1 |  | 8.8±3.5 |  | 2.3±1.2 |  | **11.2±2.5^c^** |  | 9.0±5.9 |  | 6.6±2.5 |
|  |  | **IL-4^+^** |  | 12.0±2.4 |  | 10.0±3.5 |  | 3.5±2.0 |  | **19.8±6.7^c^** |  | 10.8±4.5 |  | 10.0±5.3 |
|  |  | **IL-10^+^** |  | 51.8±13.8 |  | 49.2±16.1 |  | 56.1±22.2 |  | **130.0±45.4^c,f^** |  | 71.2±21.4 |  | **10.7±3.1^e^** |
|  |  |  |  |  |  |  |  |  |  |  |  |  |  |  |
|  |  | **CD4^+^T-cells** |  |  |  |  |  |  |  |  |  |  |  |  |
|  |  | **IFN-γ^+^** |  | 1.4±0.4 |  | 2.2±0.7 |  | 0.7±0.1 |  | **3.5±1.1^c^** |  | 2.2±1.0 |  | 2.3±1.4 |
|  |  | **IL-4^+^** |  | 1.5±0.3 |  | 1.9±0.6 |  | 1.0±0.4 |  | **2.5±0.6^c^** |  | 1.7±0.6 |  | 2.0±1.0 |
|  |  | **IL-10^+^** |  | 2.5±0.8 |  | 3.3±1.0 |  | 1.6±0.6 |  | 5.1±2.0 |  | 4.0±1.4 |  | 2.1±1.2 |
|  |  |  |  |  |  |  |  |  |  |  |  |  |  |  |
|  |  | **CD8^+^T-cells** |  |  |  |  |  |  |  |  |  |  |  |  |
|  |  | **IFN-γ^+^** |  | 0.4±0.1 |  | **1.3±0.6^a^** |  | 0.4±0.2 |  | 0.7±0.4 |  | 2.4±1.3 |  | 0.5±0.2 |
|  |  | **IL-4^+^** |  | 0.7±0.3 |  | 1.1±0.4 |  | 2.7±0.0 |  | 1.3±0.5 |  | 1.3±0.5 |  | 1.0±0.6 |
|  |  | **IL-10^+^** |  | 0.9±0.5 |  | 1.2±0.6 |  | 0.3±0.1 |  | 2.0±1.2 |  | 1.7±1.0 |  | 0.6±0.3 |
|  |  |  |  |  |  |  |  |  |  |  |  |  |  |  |

* Data are expressed as mean frequency (‰) ± standard error of cytokine^+^ cells (IFN-γ, IL-4 and IL-10) amongst gated lymphocyte subsets upon *in vitro* culture in the presence/absence of *M. leprae* antigen, including: T-cells, CD4^+^ T-cells and CD8^+^ T-cells. HHC(PB)=Household Contacts of Paucibacillary Leprosy patients; HHC(MB)=Household Contacts of Multibacillary Leprosy patients; L(PB)= Paucibacillary Leprosy patients; L(MB)= Multibacillary Leprosy patients. Multiple comparisons amongst groups were carried out by ANOVA test followed by Tuckey post-test for sequential pairwise comparisons. Additionally, Student T Test were also employed for pairwise comparative analysis. Significant differences at p<0.05 are underscored by letters “a”, “c”, “d”, “e” and “f” for comparisons to Controls, HHC(PB), HHC(MB), L(PB) and L(MB), respectively.
